# mHealth applications to enhance physical therapy outcomes among adults with chronic non-cancer pain: a scoping review

**DOI:** 10.3389/fpain.2026.1669079

**Published:** 2026-06-11

**Authors:** Katherine Beissner, Elaine Wethington, Carol Sames, Caroline Jedlicka, Lisa R. Witkin, M. Carrington Reid

**Affiliations:** 1 College of Health Professions, SUNY Upstate Medical University, Syracuse, NY, United States; 2 Department of Psychology and Department of Sociology, Cornell University, Ithaca, NY, United States; 3 Samuel J. Wood Library, Weill Cornell Medicine, New York, NY, United States; 4 Kibbee Library, Kingsborough Community College, CUNY, Brookyln, NY, United States; 5 Department of Anesthesiology, Weill Cornell Medicine, New York, NY, United States; 6 Division of Geriatrics and Palliative Medicine, Weill Cornell Medicine, New York, NY, United States

**Keywords:** chronic pain, digital apps, mHealth, physical therapy, technology

## Abstract

**Background:**

mHealth applications for managing pain in the physical therapy (PT) setting are growing in popularity. However, multiple knowledge gaps persist regarding the utility of these tools, including the types of mHealth delivery modalities tested and the amount of therapy delivered. This scoping review sought to characterize existing literature examining use of mHealth applications in adults (18+) with chronic pain eligible for PT.

**Methods:**

We searched multiple databases to identify English-language articles using pre-defined inclusion/exclusion criteria and extracted key data (e.g., study design, intervention elements).

**Results:**

Forty-two studies met eligibility criteria and were analyzed. Average participant age was 51.8 years and a substantial majority of studies (85.7%) did not report on participants’ race/ethnicity status. The mHealth interventions included stand alone apps, wearables for performance measurement (including activity trackers), and web-based content in combination with other components. Most mHealth tools delivered exercise and/or education. Most studies examined outcomes immediately or up to 3 months after completion of the intervention. The intervention results were mixed, potentially due to heterogeneity of interventions and study designs. Using the NIH Stage Model for Behavioral Intervention Development to gauge the stage of research, 64.2% of studies were classified as early-stage investigations.

**Discussion:**

This scoping review identified key knowledge gaps that can guide future research, including the need to better characterize study populations, conduct future research evaluating the impact of mHealth in older populations, conduct real world effectiveness studies, and assess both adherence to the prescribed mHealth intervention and targeted behaviors.

## 1 Introduction

Musculoskeletal conditions, including back pain, osteoarthritis, and traumatic injuries are among the most common reasons for referral to physical therapy (PT), which continues to be a first-line treatment for patients with chronic pain. Approximately 1.7 billion people worldwide are impacted by musculoskeletal conditions resulting in high rates of chronic pain, decreased physical functioning, diminished quality of life, as well as increased years lived with disability (1, 2). As populations continue to age rates of chronic pain will likely increase, leading to an expanded need for PT services.

Supporting PT interventions via remote digital technology constitutes one potential solution to meet the increased demand for PT services. mHealth tools are different from synchronous telemedicine in that interventions can be accessed through portable devices making immediate support possible with continuous monitoring using wearable devices, allowing for synchronous interactions between patients and providers, as well as asynchronous communications through notifications and alerts (3). During the initial period of the COVID-19 pandemic, many PT practices eliminated in-person appointments and markedly increased their use of these technologies. Notably, the United States experienced a 154% increase in the use of telehealth and mHealth services in March 2020 compared to the same week in 2019 (4). The development of mHealth interventions (e.g., activity trackers, mobile apps, wearable sensors, hereafter referred to as mHealth) is advancing rapidly and has substantial potential to enhance patient care and outcomes in the PT setting.

Increasing evidence suggests that delivering PT interventions using mHealth tools could improve pain and other relevant health outcomes (5, 6). mHealth can be used to record the frequency and severity of burdensome symptoms (e.g., pain, stiffness), facilitate patient-provider communication, track outcomes, monitor health behaviors (e.g., number of steps taken each day), deliver educational content, ensure exercises are performed correctly, and promote adherence to prescribed exercises as well as enhance use of other self-management techniques (5). These tools could also enhance access to care in service-poor areas, such as rural communities. Moreover, mHealth may decrease health care costs relative to conventional treatment, effectively bridging current gaps in PT treatment with respect to chronic pain management (7).

As this work expands, there is a need to evaluate how mHealth tools are being applied in various healthcare settings. Published reviews (6, 8) have reported findings on specific outcomes associated with the use of mHealth in patients undergoing PT such as pain reduction, functional activity, and quality of life, but there remains a need to assess how these tools are being employed in customary treatment settings to help patients manage diverse chronic pain conditions. Accordingly, this scoping review focuses on adults with a chronic pain condition using mHealth with a goal of characterizing: (1) the types of mHealth modalities evaluated to date (e.g., app, wearable devices, web-based) and specific content delivered via the devices (e.g., education, therapeutic exercise, pain self-management training); (2) the amount of therapy delivered including the frequency and length of the mHealth interventions; (3) whether the mHealth interventions improved pain and other study outcomes; (4) the level of patient adherence with the various mHealth interventions and whether studies examined if treatment outcomes varied as a function of level of adherence as well as other salient factors (e.g., baseline level of pain); (5) whether studies assessed for treatment effects over short, intermediate or longer-term periods of time; (6) whether mHealth tools were used independently of PT or delivered along with customary care; and finally (7) the types of studies published in this space that could range from basic science to dissemination and implementation investigations.

## 2 Methods

This study was performed following the Preferred Reporting Items for Systematic Reviews and Meta-Analyses Extension for Scoping Reviews (PRISMA-ScR) (9). The PRISMA-ScR checklist is included in the Supplementary Materials.

### 2.1 Search strategy

Upon consultation with the project team, an experienced medical librarian performed comprehensive searches to identify studies on the use and effect of mHealth for chronic pain management in PT patients. Searches were run on February 11, 2022, in the following databases: Ovid MEDLINE (ALL - 1946 to Present); Ovid EMBASE (1974 to present); CINAHL (EBSCO); and The Cochrane Library (Wiley). An updated search was run in Ovid MEDLINE (ALL - 1946 to Present) on September 22, 2024. The search strategy included all appropriate controlled vocabulary and keywords for the concepts of “chronic pain” and “mobile applications” and either “physical therapy” or “rehabilitation.” Part of the search strategy was adapted from a related Systematic Review (10). The full search strategies for all databases are available in Supplementary Appendix A. To limit publication bias there were no language, publication date, or other article type restrictions.

### 2.2 Study selection

Retrieved studies were screened for inclusion using Covidence systematic review software. Titles and abstracts were reviewed against predefined inclusion/exclusion criteria by 2 independent reviewers. Discrepancies were resolved by consensus among the research team. For final inclusion, full text was then retrieved and also screened by 2 independent reviewers. Articles were appraised for inclusion if they focused on 1) patients who were currently referred to or being treated by physical therapists or if physical therapists were involved in the design and/or delivery of the intervention, 2) adults ages 18 and above, 3) evaluated an mHealth tool in the study, 4) collected and reported patient-level data; and 5) were published in English. Excluded studies focused on child or adolescent only populations; involved patients with pain due to cancer; did not have PT involvement in the intervention; did not include an app (or a web-based intervention) that could be accessed with a cell-phone/tablet; were solely focused on synchronous telehealth; focused on treatment of acute or post-surgical pain; had no pain outcomes; were Systematic Reviews, Meta-Analyses, study descriptions, protocols, clinical trial registrations, or abstract-only publications; were non-longitudinal studies; were not published/available in English; or were published as full-text articles that could not be retrieved.

**Figure 1:**
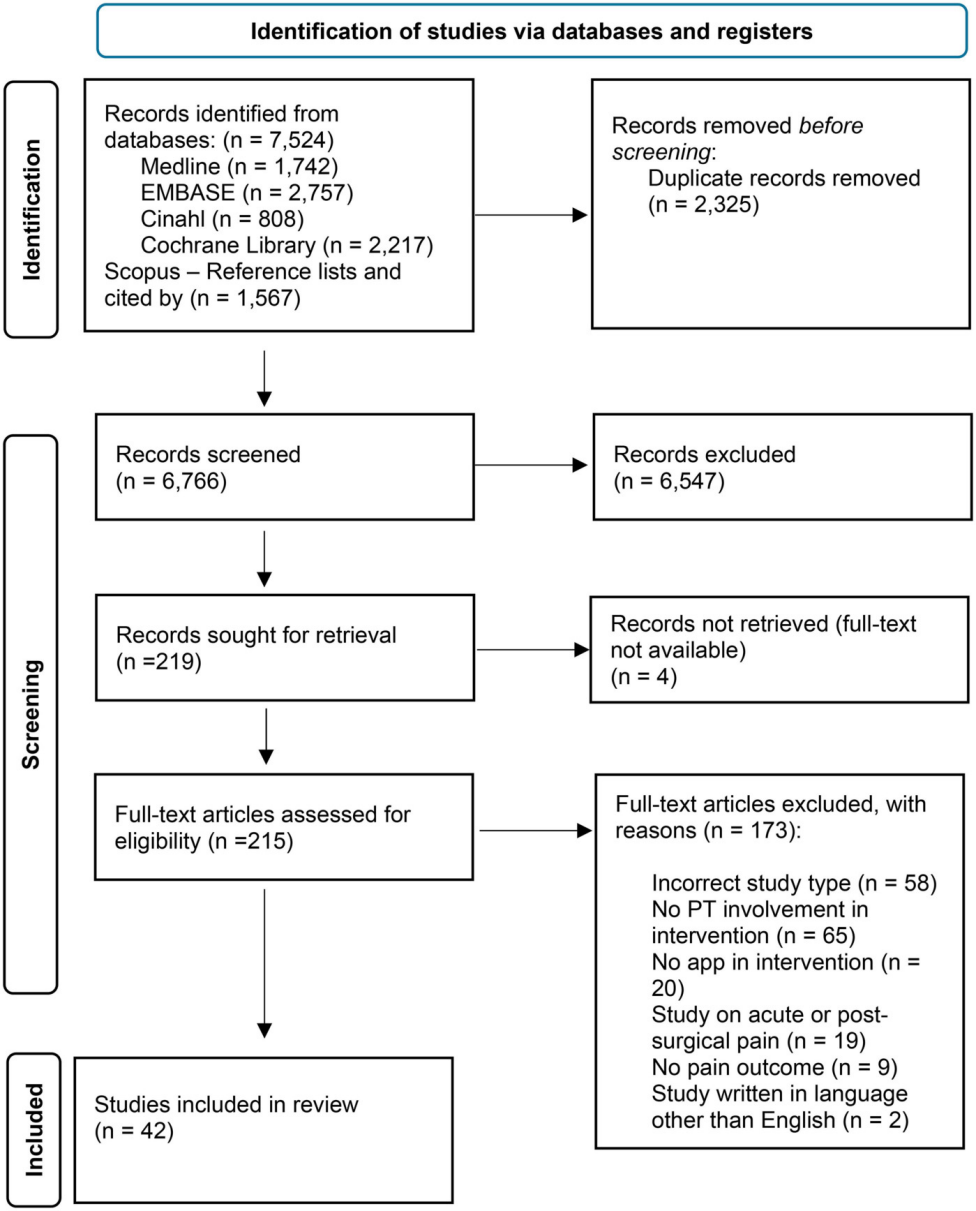
PRISMA Flow Diagram, CC-BY 4.0, Covidence.

Reference lists for the studies selected for inclusion were also pulled and searched. Figure 1 shows the full PRISMA flow diagram outlining the selection process.

### 2.3 Data extraction

Data were extracted using an extraction form developed by the authors. The data extracted included specific details to answer each study objective described above and conformed to the 5 PICOS framework components, i.e.,: (1) what types of populations were studied, (2) what types of interventions were evaluated, (3) were comparator groups included to assess for treatment efficacy, (4) what outcomes were studied, and finally (5) what types of study designs were employed. Before final analysis two authors rechecked extracted data for all studies to ensure accuracy and consistency. When disagreements arose, consensus was reached using a third reviewer. A critical appraisal of the quality of the published research was not performed as a part of this scoping review. The data were synthesized and categorized into tables generated by Excel. The variables extracted are shown in Supplementary Appendix B.

### 2.4 Study staging

We classified the included studies by stage of intervention development using the NIH Stage Model for Behavioral Intervention Development (Stages 0, basic science, Stage I, preliminary testing, Stage II-III, efficacy testing, Stage IV, effectiveness research and Stage V, dissemination and implementation research) (11). Adopting a behavioral intervention development scheme is appropriate because the primary focus of the reviewed studies was on promoting behavior change and improving lifestyle and physical wellbeing. The six stages of the model also reflect how close the intervention is to implementation in practice. Two authors initially reviewed the studies to assign stage and a third reviewed this work to confirm accuracy and resolve any disagreements in coding.

## 3 Results

Forty-two studies met the study criteria and were retained for analysis (5, 7, 12–51). The number of participants enrolled in the 42 investigations summed to 16,327.

### 3.1 Characteristics of studies

Of the 42 studies, 18 (42.9%) were conducted in Europe, 9 (21.4%) in China or other Asian countries, 6 (14.3%) in the United States, and 4 (9.5%) in Australia/New Zealand. Other studies were from Canada (3,7.1%), Africa (1, 2.4%), and the United Kingdom (1, 2.4%). The listed start and end dates of the study recruitment ranged from 2008 to 2023. Government funding sources supported 15 studies (35.7%); external foundations supported 11 (26.2%); businesses and other non-governmental organizations funded 3 (7.1%); internal funding sources were used for 2 (4.8%); while 11 articles (26.2%) did not list a funding source.

Participants were recruited in-person from PT or other healthcare practices in 29 studies (59.5%), while 4 (9.5%) also employed public media outreach. Seven studies (16.7%) used only public media as a means of recruiting participants, while 5 (11.9%) used other recruitment methods; and 1 (2.4%) did not specify a recruitment method. Table 1 summarizes the analyzed studies, including the sample size and target conditions, study location, study design and type, mHealth modality, intervention length, and intervention stage.

**Table 1:** Characteristics of studies included in the review.

Study author(s)	Study N and target conditions	Country/region of data collection	NIH stage of behavioral intervention development^a^	Study design	Type of mHealth intervention coded	Length of intervention
Alasfour et al. 2020 (12)	*N* = 40, older women with knee osteoarthritis	Other Asian	Stage II	RCT^b^	App	6 weeks
Allen et al. 2018 (13)	*N* = 35, knee osteoarthritis	United States	Stage III	RCT	Web	16 weeks
Amorim et al. 2019 (14)	*N* = 68, chronic low back pain	Australia/New Zealand	Stage Ib	RCT	App + wearable device	24 weeks
Bartholdy et al. 2019 (15)	*N* = 38, knee osteoarthritis	Europe	Stage Ib	RCT	Other	6 weeks
Bennell et al., 2020 (16)	*N* = 110, knee osteoarthritis	Australia/New Zealand	Stage IV	RCT	Other	24 weeks
Bennell et al. 2018 (17)	*N* = 144, hip osteoarthritis	Australia/New Zealand	Stage II	RCT	Web	8 weeks
Beresford et al. 2022 (5)	*N* = 814, musculo-skeletal problems (any site or cause)	United States	Stage 0	Cohort study	App	Average=9.6 weeks (range 8.8–10.4)
Chen et al. 2020 (18)	*N* = 15, shoulder adhesive capsulitis	Other Asian	Stage Ia & Stage Ib (two trials)	Non-randomized experimental study	App + wearable device	12 weeks
Cronstrom et al. 2019 (19)	*N* = 458, patients post osteoarthritis treatment program	Europe	Stage 0	Cohort study	Web	6 weeks
Gardner et al. 2022 (20)	*N* = 99, chronic pain (site and source not specified)	Australia/New Zealand	Stage Ib	RCT	Web	16 weeks
Geraghty et al. 2018 (21)	*N* = 87, low back pain	United Kingdom	Stage Ib	RCT	Web	6 weeks
Hörder et al. 2022 (22)	*N* = 2,593, low back pain, sub-acute or chronic	Europe	Stage 0	Cohort study	App	12 weeks
Janela et al. 2022 (23)	*N* = 132, elbow pain	United States	Stage 0	Non-randomized clinical trial	App + wearable device	8 weeks
Juhlin et al. 2021 (24)	*N* = 138, chronic widespread pain	Europe	Stage III	RCT	Web	24 weeks
Kaufman et al. 2021 (7)	*N* = 345, knee osteoarthritis	United States	Stage III	RCT	Web	36 weeks
Kim et al. 2024 (25)	*N* = 34, non-specific neck pain	Other Asian	Stage Ib	RCT	App	6 weeks
Kloek et al. 2018 (26)	*N* = 123, hip or knee osteroarthritis	Europe	Stage III	RCT	Web	12 weeks
Kloek et al. 2019 (27)	*N* = 41, non-specific back pain	Europe	Stage 1b	Non-randomized experimental study	Web	12 weeks
Krkoska et al. 2023 (28)	*N* = 27, chronic non-specific low back pain	Europe	Stage Ib	Non-randomized experimental study	App	18 weeks
Lara-Palomo et al. 2022 (29)	*N* = 74, chronic lower back pain	Europe	Stage IV	RCT	Web	8 weeks
Li et al. 2017 (32)	*N* = 34, knee osteoarthritis	Canada	Stage Ib	RCT	Other	4 weeks
Li et al. 2020a (30)	*N* = 118, inflammatory arthritis	Canada	Stage III	RCT	App + wearable device	27 weeks
Li et al. 2020b (31)	N - 51, knee osteoarthritis	Canada	Stage II	RCT	App + wearable device	12 weeks
Lopez-Marcos et al. 2024 (33)	*N* = 98, chronic nonspecific low back pain	Europe	Stage III	RCT	App	12 weeks
Mbada et al. 2019 (34)	*N* = 49, chronic low back pain	Africa	Stage III	RCT	App	8 weeks
Nordin et al. 2016 (35)	*N* = 99, chronic pain or generalized pain	Europe	Stage Ib	RCT	Web	16 weeks
Özden et al. 2022 (36)	*N* = 50, chronic low back pain	Europe	Stage 1b	RCT	Web	8 weeks
Özel et al. 2022 (37)	*N* = 71, chronic nonspecific neck pain	Europe	Stage Ib	RCT	Web	4 weeks
Rafiq et al. 2021 (38)	*N* = 114, overweight or obese patients with knee osteoarthritis	South Asia (India/ Pakistan/Bangladesh)	Stage III	RCT	Other	12 weeks
Raiszadeh et al. 2021 (39)	*N* = 1090, low back pain	United States	Stage III	Non-randomized experimental study	Web	12 weeks
Ramalingam et al. 2023 (40)	*N* = 15 male athletes with ankle pain	Other Asian	Stage Ib	RCT	App	6 weeks
Rodriguez Sanchez-Laulhe et al. 2022 (42)	*N* = 69, rheumatism in hand	Europe	Stage III	RCT	App	12 weeks
Rodriguez Sanchez-Laulhe et al. 2023 (41)	*N* = 74, symptomatic hand osteoarthritis	Europe	Stage III	RCT	App	12 weeks
Sandal et al. 2021 (43)	*N* = 461, non-specific low back pain	Europe	Stage IV	RCT	App + wearable device	12 weeks
Scheer et al. 2022 (44)	*N* = 6,949, musculo-skeletal pain	United States	Stage 0	Cohort study	App + wearable device	12 weeks
Shi et al. 2024 (45)	*N* = 54, nonspecific low back pain	China	Stage II	RCT	App + wearable device	8 weeks
Toelle et al. 2019 (46)	*N* = 101, low back pain	Europe	Stage Ib	RCT	App	12 weeks
Uesugi et al. 2018 (47)	*N* = 46, hip osteoarthritis	Other Asian	Stage Ib	Non-randomized experimental study	Web	12 weeks
Weber et al. 2024 (48)	*N* = 50, hip or knee osteoarthritis	Europe	Stage Ib	RCT	App	12 weeks
Weise et al. 2022 (49)	*N* = 213, unspecific and degenerative back pain	Europe	Stage II	RCT	App	12 weeks
Yang et al. 2019 (50)	*N* = 8, low back pain	China	Stage Ib	RCT	App	4 weeks
Zheng et al. 2022 (51)	*N* = 37, nonspecific chronic low back pain	China	Stage Ib	RCT	App	4 weeks

a Stage 0 = basic science, Stage Ia = reserch focused on modifying, adapting, or refining an existing intervention, Stage 1b=pilot and feasibility testing; Stage II = customary efficacy testing, Stage III=real world efficacy testing and Stage IV = effectiveness.

research.

b RCT, randomized controlled trial.

### 3.2 Characteristics of the study populations

Across all studies, the average participant age was 51.8 years. In 13 studies (31.0%), participants had a mean age between 60 and 70 years, 11 (26.2%) reported a mean age between 50 and 59, 13 (31.0%) reported a mean age in the 40–49-year old range, and the remaining 5 (11.9%) enrolled subjects who were, on average, in their twenties or thirties. Of the 41 studies (97.6%) reporting participant gender, the mean percentage of female subjects was 61.2%. A substantial majority of studies (36, 85.7%) did not report data on participants' race/ethnicity status. Of note, only 3 of the 6 studies conducted in the US reported race/ethnicity data. Twenty-one studies (50%) reported participants' education level.

The primary inclusion criterion was chronic pain. Other inclusion criteria included pain location, specified in 37 studies (88.1%) and diagnosis (19, 45.2%), with 16 (38.1%) specifying both a diagnosis and pain location (e.g., knee osteoarthritis). Two studies (4.8%) specified subject gender/sex criteria, and 5 (11.9%) specified a level of pain-related disability. Primary study exclusion criteria included neurological conditions including cerebrovascular disease, traumatic brain injury or other (19, 45.2%); dementia (11, 26.2%), cardiopulmonary conditions (11, 26.2%), recent trauma or surgery (19, 45.2%), and pregnancy (10, 23.8%). Other specific excluded diagnoses were included in 22 studies (52.4%). Primary study question results are presented below.

### 3.3 Specific types of mHealth studied and associated content

The various mHealth interventions were delivered using a range of modalities, including stand-alone apps, apps with other components (e.g., a wearable or external device for performance measurement, including activity trackers), web-based content delivery, or web-based content in combination with other components. One study (2.4%) provided support and educational content solely through text messages. The most common mHealth content was exercise: A total of 34 studies (81.0%) included exercise with 20 (47.6%) also providing some type of education. Table 2 shows the type of mHealth content delivered as a function of intervention modality.

**Table 2:** mHealth content by delivery method.

	mHealth Content			
Type of mHealth	Exercise	Exercise and Education	Education	Total
Web	5 (11.9%)	8 (19.0%)	2 (4.8%)	15 (35.7%)
App	7 (16.7%)	8 (19.0%)		15 (35.7%)
App+wearable device	5 (11.9%)	3 (7.1%)	1 (2.4%)	9 (21.3%)
Other^a^	1 (2.4%)	1 (2.4%)	1 (2.4%)	3 (7.1%)
Total	18 (42.9%)	20 (47.6%)	4 (9.5%)	42 (100%)

a Other mHealth type includes texting, including texting with activity trackers and web content.

Twenty-seven studies (64.3%) used mHealth to guide progression of exercise, 18 (42.9%) tailored the exercises to each individual user, while the remaining 9 (21.4%) used standardized exercise progressions. In 17 studies (40.5%) the mHealth intervention allowed for patient-provider communication using text, app, or online modalities.

### 3.4 Effectiveness of the mHealth tools

The evaluation of intervention effectiveness was complicated by the lack of comparison groups in 7 studies (16.7%). Another 5 studies (11.9%) included the mHealth intervention in multiple arms of the trial, examining the impact of adding other interventions such as coaching, physical therapy, or an additional mHealth module on study outcomes. Less than half of the studies (18, 42.8%) specifically tested for minimally clinically important differences (MCID).

While in general patients improved in pain and pain-related disability over the course of the intervention, 14 (33.3%) studies conducted between-group comparisons evaluating the impact of mHealth vs. other care modalities and found greater positive impact on pain and pain-related disability. To distinguish between statistically significant differences and clinically important differences we used a decrease of 2 points in the 11-point numerical pain rating scale to indicate a MCID (52), and standards identified elsewhere for condition specific measures (53–55). Eleven studies (26.2%) showed improvement above the MCID for at least one measurement instrument. Of these, 7 (63.6%) were app-based, 2 (18.2%) used apps and a wearable device, 1 (9.1%) was web-based, and 1 (9.1%) used text messaging.

In 6 (54.5%) of the studies with change in pain outcomes above the MCID the interventions lasted 12 weeks, 2 (18.2%) lasted 4 weeks, 2 (18.2%) lasted 6 weeks, and 1 (9.1%) was 27 weeks. All but one of the studies with clinically meaningful change included exercise as part of the mHealth intervention, with 2 (18.2%) also including communication, 4 (36.4%) including education, and 1 (9.1%) including both education and communication alongside the exercise.

Seven studies (16.7%) compared mHealth-delivered exercise and education to written instructions and demonstrated a superior outcome in at least one pain-related measure in the mHealth groups, with three (42.9%) showing clinically meaningful differences. Two studies (28.6%) that compared mHealth to in-person PT treatment showed non-significant between-group differences in outcomes, and one (14.3%) showed clinically significant improvement in pain-related disability in the mHealth group, a finding not seen in the usual care group.

Thirteen studies incorporated coaching either within or alongside the mHealth intervention. Eight of these studies (61.5%) showed superior results compared to the control group in at least one clinical measure.

### 3.5 Intensity of therapy delivered

The length of the mHealth interventions varied considerably, lasting 4 to 6 weeks in 11 studies (26.2%), between 7 and 12 weeks in 17 (40.5%), from 13 to 24 weeks in 10 (23.8%), and 25 or more weeks in the remaining 4 studies (9.5%). The intended frequency of participant interaction with the mHealth programs also varied considerably from daily in 14 studies (33.3%), to 3 or 4 times per week in 9 (21.4%), to weekly in 4 (9.5%) studies. The remaining 9 studies (21.4%) asked participants to use the device on an as-needed basis or some other interval. Five studies (11.9%) reported different frequencies of interaction for different components of the mHealth content, most commonly exercise daily and education weekly. One study (2.4%) did not specify the intended frequency.

### 3.6 Level of intervention adherence

Thirty-three studies (78.6%) reported that intervention adherence was measured. Twenty-five studies (75.8%) assessed adherence to mHealth-delivered treatment using an app or web-based data collection method, most by a count of logins to the digital platform; 3 (9.1%) also incorporated self-reported adherence. Six studies (18.2%) relied solely on self-report and 2 (6.1%) did not report the method used to assess adherence. Sixteen investigations (48.5%) tracked adherence to the mHealth protocol, 9 (27.3%) reported on participant adherence to a prescribed exercise or physical activity program, 6 (18.2%) reported adherence to both mHealth content and an exercise or physical activity program, and 1 (3.0%) did not specify whether adherence measures tracked use of mHealth or a prescribed exercise regimen. The remaining study reported adherence to an in-person component of the intervention. High levels of program adherence (over 70%) were reported in 8 studies (24.2%), 9 (27.3%) reported a level of adherence of 30%–70%, 6 (18.2%) reported adherence in vague terms, and the remaining 10 (30.3%) did not report adherence data.

A minority of studies (7, 16.7%) examined the relationship between adherence and study outcomes. Of these, 6 (85.7%) reported that higher program adherence was associated with improved study outcomes and 1 (14.3%) found no relationship between adherence and outcomes.

### 3.7 Other issues related to adherence

Three studies (9.1%) reported that inclusion of practitioner interaction was associated with higher program adherence. Insufficient data were provided to compare level of adherence as a function of mHealth modality. We also searched to determine whether studies reported on adherence with tool use over time. Only 2 studies (4.8%) did this; both reported that adherence declined over the intervention period.

### 3.8 Length of follow-up

The majority of studies assessed outcomes immediately after completion of the intervention (47.6%) or up to 3 months after intervention completion (21.4%). Eight studies (19.1%) assessed outcomes between 4 and 12 months after completion of the intervention period, while 5 (11.9%) also assessed outcomes 12 or more months after program completion.

### 3.9 Were mHealth tools used independently of PT or delivered along with customary care?

We examined the types of co-therapies delivered alongside the mHealth intervention. In 14 studies (33.3%) the mHealth component of the intervention was delivered along with usual care. The remaining 28 studies (67.6%) provided one or more additional treatments concurrent with the mHealth intervention. Of the 35 studies that incorporated control groups, 3 (8.6%) provided no treatment to participants in the control arm and 4 (11.4%) employed a waitlist control design. The treatments provided to both groups are shown in Table 3.

**Table 3:** Additional treatments provided, by group.

Additional Interventions Provided	mHealth Group *n* = 42	Control Group *n* = 35
Physical Therapy^a^	6 (14.3%)	11 (31.4%)
Home Exercise Program	6 (14.3%)	7 (20.0%)
Education	4 (9.5%)	8 (22.9%)
General Physical Activity	7 (16.7%)	3 (8.6%)
Coaching	8 (19.0%)	1 (2.9%)
Other	2 (4.8%)	3 (8.6%)
Pain Modalities	2 (4.8%)	2 (5.7%)
None	14 (33.3%)	3 (8.6%)
Waitlist		4 (11.4%)

7 studies did not have a control group.

a 1 study provided PT virtually.

### 3.10 Stage of intervention development

Most of the included studies were classified as early-stage research. Of the 42 studies, 5 (11.9%) were categorized as Stage 0 (basic science) examining behavioral mechanisms prior to developing the actual intervention. The largest proportion of studies (19; 45.2%) involved Stage I activities, which included preliminary testing of a new intervention, modification and/or adaptation of an existing intervention, or testing the feasibility and acceptability of a mHealth intervention in a population of adult patients with chronic pain. Four articles (9.5%) were classified as Stage II efficacy studies (experimental testing with researchers delivering the mHealth intervention to participants), while 11 (26.2%) were Stage III efficacy studies (experimental testing in community settings delivered by community providers, including PTs). Three studies (7.1%) were classified as Stage IV effectiveness studies, based in clinical or PT practices implementing procedures to maximize external validity. There were no published Stage V studies identified in our search that tested or examined strategies of implementation or adoption in PT or other clinical practices.

Our purpose for classifying studies by the NIH Stage Model was to assess how close scientifically rigorous mHealth tools supporting PT are to dissemination to patients and providers, implementation in practice, and to commercialization (11). Of the 42 reviewed studies, 24 (57.1%) reported developing original apps and web-based interventions, while 18 (42.9%) tested, adopted, or adapted pre-existing mHealth intervention tools that were available via app stores or websites. Among the 18 studies testing pre-existing tools, we found 4 (22.2%) that were developing or evaluating tools already promoted or being adopted by government-based health institutions as models of care. In addition, we found (using Google search) that one of the newly-developed mHealth tools may currently be undergoing commercialization; however, evidence of new commercialization may be an under-estimate of the rate of implementation of mHealth tools into PT practice.

### 3.11 Other relevant findings

#### 3.11.1 Outcome measures

All studies incorporated a measure of pain intensity. Other pain measures included pain impact (15 studies, 35.7%), and frequency (6, 14.3%). Instruments used to measure pain included the Numeric Rating Scale (24, 57.1%), the Visual Analog Scale (7, 16.7%), joint/region or diagnosis-specific tools (15, 35.7%), and various disability instruments (15, 35.7%). The majority of studies used multiple pain instruments (23, 54.8%)

Of the 25 studies (59.5%) that assessed psychosocial constructs through self-report (PSC-SR), 10 (40.0%) assessed self-efficacy, while 8 (32.0%) measured quality of life. Other PSC-SR assessed in the analyzed studies included measures of fear avoidance or kinesiophobia 7 (28.0%), depression and/or anxiety 5 (20.0%), and other measures (8, 32.0%). Seventeen studies (28.0%) did not include PSC-SR outcomes.

#### 3.11.2 Potential moderators of treatment effect

Nine (21.4%) studies assessed for other possible moderators of treatment effect besides adherence with the mHealth tool to include level of pain at study entry (3, 33.3%), and 1 study (11.1%) each for the following potential moderators: pain duration, age, gender, disease type, functional status, and race/ethnicity.

#### 3.11.3 Losses to follow-up

A substantial majority of studies 36 (85.7%) reported on losses to follow-up. Eight of these investigations (22.2%) did not report reasons for the losses. The remaining 28 investigations cited specific reasons; 6 (21.4%) cited reasons related to the mHealth tool under investigation. Thirty-six individuals were listed as a drop out because of limited use of the mHealth tool or having stopped use of the device altogether, while 5 individuals were cited as having dropped out due to technical issues related to device use.

## 4 Discussion

This scoping review characterized the current literature addressing the use of mHealth tools designed to help PT providers treat adult patients suffering with chronic pain.

### 4.1 Types of mHealth studied

This study documents that a wide range of mHealth tools have been evaluated as tools to enhance pain management in PT settings, including stand-alone apps, apps with other components (e.g., use of a wearable device), web-based content delivery, or web-based content in combination with other components. As mHealth tools continue to develop, we can anticipate the testing of devices that can generate even more sophisticated types (and amounts) of data for use by both providers and patients. It will be important for future research to define what data elements are valued most by these 2 end-user groups. Further, available research indicates that adoption of mHealth apps on the part of patients and providers in healthcare setting remains low (56–59). Development of new tools that employ user-centered designs in the development process, a process which prioritizes the needs, preferences, and challenges of end-users throughout the development process, is likely to pay dividends with respect to patient and provider adoption. Finally, we did not identify any investigations that conducted head-to-head comparisons of two or more mHealth tools but encourage future research in this area.

### 4.2 Types of mHealth content

Since this review was focused on PT applications of mHealth it is not surprising that we found that exercise was the most common mHealth content. Exercise was most often combined with educational elements, with a small minority delivering educational elements alone. This is consistent with a recent analysis of mHealth content delivered by commercially available apps, though Zhou and colleagues found fewer apps geared toward individuals with low back pain incorporated education alongside exercise compared to apps for other conditions (60). Given that our study focused on individuals eligible for PT, it is also not surprising that a majority of studies in this review employed an educational component as part of their mHealth intervention. This finding is consistent with current PT clinical practice guidelines (CPGs) for patients with low back pain which recommend education for all levels of acuity—acute, chronic, and post-operative (61), while both education and exercise are included in Danish CPGs for patients with knee or hip osteoarthritis (62).

### 4.3 Length of interventions

There was considerable variability in the length of time participants were asked to interact with a given mHealth intervention. This, combined with the poor level of detail regarding the intended frequency of mHealth use in some studies, raises questions about the true dose of the intended interventions.

### 4.4 Timing of outcome assessments

The timing of outcome assessments also varied greatly, from immediate post-intervention to 6 months or longer. If the intent of mHealth interventions is health behavior change (e.g., incorporating a set of exercises into a daily or weekly routine), an extended follow-up period seems appropriate, but this has not been consistently applied. One notable exception was a study that used a web-based intervention to deliver exercises for patients with lower extremity osteoarthritis, incorporating long-term follow-up to measure impact.

### 4.5 Adherence assessments

We sought to examine adherence to mHealth interventions and found that while a substantial majority of studies (33, 78.6%) measured adherence, not all explicitly reported adherence rates. There is some indication that integrating practitioner follow-up through phone calls, chats or texts is associated with increased adherence to mHealth interventions (17, 19, 21, 28, 31, 32), but others with high adherence did not incorporate this type of interaction (43, 49). One study not included in this review (63) analyzed data collected from a study that was included (13) to examine the relationship between program engagement and pain outcomes. Greater utilization of in-person PT was associated with improved outcomes, but this did not hold true for the mHealth intervention. These results suggest that developing a personal relationship with PT practitioners enhances outcomes, a challenge for future implementation of mHealth interventions. These findings further highlight the need for improved reporting of adherence measures and associated outcomes in future research.

### 4.6 Outcome measures

All studies included in this review incorporated a measure of pain intensity, typically an 11-point numerical rating scale, either alone or as part of a larger pain inventory. These self-report scales are considered the gold standard for pain intensity (64). Well-validated condition or region-specific instruments such as the Oswestry Disability Index (65), Roland Morris Disability Questionnaire (66), Western Ontario and McMaster Universities Osteoarthritis Index (WOMAC) (67), and the Knee Injury and Osteoarthritis Outcome Score (KOOS) (68) were also employed.

These measures rely on participants' self-report of pain, function and impact rather than relying on performance-based measures of strength, mobility, and/or flexibility. Measurement of physical abilities requires a degree of clinical expertise on the part of the research team, as well as access to a testing site for study subjects. Some studies included in this review incorporated performance measures such as the Timed Up and Go (69, 70) and the Short Physical Performance Battery (71, 72) which are commonly used in PT outcomes research and have established validity and reliability. Comparisons of in-person therapy to mHealth-augmented care rightly incorporates this type of measurement.

### 4.7 Stage of intervention development

A substantial majority (28, 66.7%) of the analyzed studies were classified as being at an early stage of intervention development, i.e., were classified as at either Stage 0, I, or II, with 19 (45.2%) reporting intervention development activities only, e.g., assessing feasibility and acceptability of a given intervention. These proportions reflect a common situation in intervention research, that developmental research often requires refinement before the most promising interventions can be implemented into practice after its effectiveness is established.

While most studies in our sample were classified as early-stage research, the smaller proportion of higher stage studies may be a conservative estimate of the penetration of mHealth tools into PT practice. Moreover, the use of commercially available products in interventions may result in more rapid development and implementation of efficacious care programs that include mHealth tools (30–32).

### 4.8 mHealth effectiveness

The intervention effectiveness results were mixed and given the heterogeneity of interventions and study designs it is difficult to ascertain what aspects of mHealth intervention are most impactful in promoting meaningful clinical change. For example, while some studies showed superior results when coaching was included with the mHealth intervention (5, 14, 19, 22–24, 30, 31, 37), others that included this type of interaction showed no differences from the control group (20, 21, 26, 45). This is consistent with a prior systematic review of coaching by physical therapists which found varied impact of coaching (73). Thus, while coaching has been shown to have a positive impact on pain and disability for some patients (74), further research in this area is warranted to identify optimal mHealth coaching content and parameters.

The majority of the studies that demonstrated clinically meaningful improvements had a relatively long intervention period, 12 or more weeks, suggesting that sustained use of mHealth over time may reap more benefits. The lack of a pattern of type of mHealth delivery (app, web or wearable), or mHealth content is perhaps reflective of the flexibility of mHealth platforms for conveying meaningful content.

### 4.9 Other relevant findings - study population characteristics

The demographics of the study samples were representative of the wider chronic pain population. In studies that reported gender, most were female. In addition, participants were middle- aged with most participants with mean ages in the 40's-60's. None, however, had a mean age of 70 or higher, thus missing valuable guidance on the use of mHealth for this older population.

Half of the studies reported the educational background of study subjects, while only 6 (14.3%) reported on the race/ethnicity of the sample. In the United States there is a strong body of research that shows differences in treatments provided to patients from minoritized backgrounds as compared to non-Hispanic White individuals, and those with higher levels of education also receive more or better treatment than those with lower education (75, 76). Given these disparities in care, it seems prudent to consider whether potential beneficial mHealth interventions are delivered equitably across patient populations.

### 4.10 Implications for research

Our scoping review identified substantial heterogeneity with respect to significance testing. While guidelines (77) call for determining whether clinically meaningful differences are associated with a given intervention, only 18 (42.8%) of the studies in our sample did so. Benchmarks for identifying clinically important changes in pain outcome measures have been published and are recommended for use in future investigations (77). Further, our study documents a paucity of testing for heterogeneity with respect to treatment effects. Only 1 in 5 studies assessed for the possibility of effect modification regarding factors such as race/ethnicity, age, gender, and level of pain at the time of study entry. Future studies should examine how treatment benefits vary across important patient subgroups, moving beyond average results to inform personalized medicine.

This review also documented substantial variability in the reporting of results. For example, a substantial majority of the analyzed studies failed to report on the race/ethnicity status of their samples. Further, one in five studies failed to report losses to follow-up or failed to cite the reasons for the losses. Future investigations should use customary reporting guidelines [e.g., 2025 CONSORT checklist (78)] when reporting results. Additional guidelines relevant to the publication of clinical trial results focused on pain (79) and the evaluation of mHealth devices (80) have been published and should be considered as well.

Our study has identified important gaps that can serve as a roadmap for future research in this area. Our research confirms that existing research has focused on evaluating mHealth tools for use by middle-aged and “young-old” adults with chronic pain. Given that older adults ages 75 and above frequently undergo PT on account of various chronic pain problems, future research is needed to assess the feasibility, acceptability and efficacy of these tools in this expanding patient population. Our study identifies an additional gap with respect to the infrequent reporting of race/ethnicity data. These data are critical to provide in published reports so that practitioners can assess the generalizability of study findings and make evidence-informed decisions. Investigators should also be reporting treatment differences as a function of race/ethnicity subgroups when possible. Our study also documents that few published reports (fewer than 1 in 5) achieved high levels of adherence (over 70%) with the mHealth tools being studied. It will be important to develop and test strategies to enhance adherence as a means of optimizing treatment outcomes in this target population. Prior research has identified that individualized push notifications (e.g., personalized text messages), use of mHealth tools that have been developed via user-centered design principles, and offering tech support have all been shown to enhance adherence to digital tools (81). Finally, relatively few studies in this review have advanced beyond the efficacy stage of intervention development, making meaningful conclusions about an intervention's effectiveness and performance under “real-world” conditions difficult. More pragmatic trials along with studies focused on implementing mHealth tools into customary PT practice are clearly needed.

### 4.11 Implications for practice

Our review documented a wide range of adherence rates across the various mHealth devices tested, often with a lack of detail on adherence measurement. However, it appears that uptake of mHealth interventions may be enhanced by personal contact with a physical therapist through in person sessions (33), coaching phone calls (21), or even by text reminders (17). Therefore, when incorporating mHealth into a patient's treatment program, it is important to follow up to encourage sustained mHealth use and troubleshoot any problem areas or barriers to use. If using mHealth to encourage the completion of a home exercise program or physical activity plans after discharge from PT, incorporating text reminders or occasional phone or virtual check-ins will likely enhance adherence and prolong the benefit of therapy.

The proliferation of mHealth tools will only increase in the coming years. Clinicians struggle to keep up with these advances (82). A recent review analyzed existing frameworks for evaluating these tools (83); use of such frameworks can help clinicians make informed decisions about which tools may be appropriate for their respective practices. Further, digital libraries that evaluate and provide data on the safety, effectiveness and quality of mHealth devices are becoming available to clinicians and patients. The Organization for the Review of Care and Health Apps (ORCHA) has one of the largest libraries that is readily accessible to both providers and patients. This site has reviewed thousands of apps against 300 + criteria that rate apps for their safety, efficacy, privacy and usability (https://www.orchahealth.com/, accessed 04/20/2026). Clinicians should consider using ORCHA (as well as other digital libraries) when making decisions about whether to incorporate a given mHealth tool into practice.

Finally, while mHealth technologies are likely to provide substantial benefit to both patients and PT providers in the coming decades, such advances will also come with substantial challenges. Clinicians should be mindful that these tools have the potential to worsen existing health inequities, given established disparities in access to technology and connectivity.

### 4.12 Study limitations

There are several limitations to this review that merit consideration. Some tech-based studies may not be published in MEDLINE-indexed journals which were the foundation of this review. In addition, we excluded non-English language publications and likely missed relevant work published in other languages. By excluding studies addressing pain in adolescent and pediatric populations we eliminated work that may have application to adult populations. We did not assess the quality of the studies, focusing instead on the range of work being done in this area. Finally, we did not evaluate studies to determine whether they assessed for possible intervention-associated harms but encourage future research in this area to quantify both the benefits as well as potential harms of mHealth applications in populations receiving PT.

## 5 Conclusions

This scoping review documents the promise of mHealth as tools that have the potential to measurably enhance PT outcomes among adults with chronic pain. Addressing the key knowledge gaps identified in this study could help to advance our understanding of the role of mHealth in the PT setting. To advance knowledge in this space more research is needed, specifically in the areas of understudied populations (e.g., conducting intervention studies on older-old adults), testing strategies to enhance mHealth tool adherence, and conducting more “real-world” effectiveness trials with extended follow-up to ascertain whether benefits sustained over the short-term persist over time.

## Data Availability

The raw data supporting the conclusions of this article will be made available by the authors, without undue reservation.

## References

[1] Musculoskeletal Health. (2022). Available online at: https://www.who.int/news-room/fact-sheets/detail/musculoskeletal-conditions (Accessed April 21, 2026)

[2] Cieza A Causey K Kamenov K Hanson SW Chatterji S Vos T. Global estimates of the need for rehabilitation based on the global burden of disease study 2019: a systematic analysis for the global burden of disease study 2019. Lancet. (2021) 396(10267):2006–17. 10.1016/S0140-6736(20)32340-0 33275908 PMC7811204

[3] Thompson D Rattu S Tower J Egerton T Francis J Merolli M. Mobile app use to support therapeutic exercise for musculoskeletal pain conditions may help improve pain intensity and self-reported physical function: a systematic review. J Physiother. (2023) 69(1):23–34. 10.1016/j.jphys.2022.11.012 36528508

[4] Koonin LM Hoots B Tsang CA Leroy Z Farris K Jolly T. Trends in the use of telehealth during the emergence of the COVID-19 pandemic - United States, January-march 2020. MMWR Morb Mortal Wkly Rep. (2020) 69(43):1595–9. 10.15585/mmwr.mm6943a3 33119561 PMC7641006

[5] Beresford L Norwood T. The effect of Mobile care delivery on clinically meaningful outcomes, satisfaction, and engagement among physical therapy patients: observational retrospective study. JMIR Rehabil Assist Technol. (2022) 9(1):e31349. 10.2196/31349 35107436 PMC8851343

[6] Moreno-Ligero M Moral-Munoz JA Salazar A Failde I. Mhealth intervention for improving pain, quality of life, and functional disability in patients with chronic pain: systematic review. JMIR Mhealth Uhealth. (2023) 11:e40844. 10.2196/40844 36729570 PMC9936365

[7] Kaufman BG Allen KD Coffman CJ Woolson S Caves K Hall K. Cost and quality of life outcomes of the stepped exercise program for patients with knee osteoarthritis trial. Value Health. (2022) 25(4):614–21. 10.1016/j.jval.2021.09.018 35365305

[8] Rintala A Rantalainen R Kaksonen A Luomajoki H Kauranen K. Mhealth apps for low back pain self-management: scoping review. JMIR Mhealth Uhealth. (2022) 10(8):e39682. 10.2196/39682 36018713 PMC9463614

[9] Tricco AC Lillie E Zarin W O'Brien KK Colquhoun H Levac D. PRISMA Extension for scoping reviews (PRISMA-ScR): checklist and explanation. Ann Intern Med. (2018) 169(7):467–73. 10.7326/M18-0850 30178033

[10] Niknejad B Bolier R Henderson CR Jr Delgado D Kozlov E Löckenhoff CE. Association between psychological interventions and chronic pain outcomes in older adults: a systematic review and meta-analysis. JAMA Intern Med. (2018) 178(6):830–9. 10.1001/jamainternmed.2018.0756 29801109 PMC6145761

[11] Onken LS Carroll KM Shoham V Cuthbert BN Riddle M. Reenvisioning clinical science: unifying the discipline to improve the public health. Clin Psychol Sci. (2014) 2(1):22–34. 10.1177/2167702613497932 25821658 PMC4374633

[12] Alasfour M Almarwani M. The effect of innovative smartphone application on adherence to a home-based exercise programs for female older adults with knee osteoarthritis in Saudi Arabia: a randomized controlled trial. Disabil Rehabil. (2020) 44(11):2420–7. 10.1080/09638288.2020.1836268 33103499

[13] Allen KD Arbeeva L Callahan LF Golightly YM Goode AP Heiderscheit BC. Physical therapy vs internet-based exercise training for patients with knee osteoarthritis: results of a randomized controlled trial. 2018;(no pagination). 10.1016/j.joca.2017.12.008 PMC6021028 29307722

[14] Amorim AB Pappas E Simic M Ferreira ML Jennings M Tiedemann A. Integrating Mobile-health, health coaching, and physical activity to reduce the burden of chronic low back pain trial (IMPACT): a pilot randomised controlled trial. BMC Musculoskelet Disord. (2019) 20(1):71. 10.1186/s12891-019-2454-y 30744606 PMC6371593

[15] Bartholdy C Bliddal H Henriksen M. Effectiveness of text messages for decreasing inactive behaviour in patients with knee osteoarthritis: a pilot randomised controlled study. Pilot Feasibil Stud. (2019) 5(101676536):112. 10.1186/s40814-019-0494-6 PMC6732192 31516729

[16] Bennell KL Nelligan RK Rini C Keefe FJ Kasza J French S. Effects of internet-based pain coping skills training before home exercise for individuals with hip osteoarthritis (HOPE trial): a randomised controlled trial. Pain. (2018) 159(9):1833–42. 10.1097/j.pain.0000000000001281 29794609

[17] Bennell K Nelligan RK Schwartz S Kasza J Kimp A Crofts SJ. Behavior change text messages for home exercise adherence in knee osteoarthritis: randomized trial. J Med Internet Res. (2020) 22(9):e21749. 10.2196/21749 32985994 PMC7551110

[18] Chen YP Lin CY Tsai MJ Chuang TY Lee OKS. Wearable motion sensor device to facilitate rehabilitation in patients with shoulder adhesive capsulitis: pilot study to assess feasibility. J Med Internet Res. (2020) 22(7):e17032. 10.2196/17032 32457026 PMC7413285

[19] Cronström A Nero H Dahlberg LE. Factors associated with Patients’ willingness to consider joint surgery after completion of a digital osteoarthritis treatment program: a prospective cohort study. Arthritis Care Res (Hoboken). (2019) 71(9):1194–201. 10.1002/acr.23772 30298990 PMC6771662

[20] Gardner T Schultz R Haskelberg H Newby JM Wheatley J Millard M. The effect of adjunct telephone support on adherence and outcomes of the reboot online pain management program: randomized controlled trial. J Med Internet Res. (2022) 24(2):e30880. 10.2196/30880 35113021 PMC8855305

[21] Geraghty AWA Stanford R Stuart B Little P Roberts LC Foster NE. Using an internet intervention to support self-management of low back pain in primary care: findings from a randomised controlled feasibility trial (SupportBack). BMJ open. (2018) 8(3):e016768. 10.1136/bmjopen-2017-016768 29525768 PMC5879455

[22] Hörder H Nero H Misini Ignjatovic M Kiadaliri A Lohmander LS Dahlberg LE. Digitally delivered exercise and education treatment program for low back pain: longitudinal observational cohort study. JMIR Rehabil Assist Technol. (2022) 9(2):e38084. 10.2196/38084 35727622 PMC9257621

[23] Janela D Costa F Molinos M Moulder RG Lains J Bento V. Digital rehabilitation for elbow pain musculoskeletal conditions: a prospective longitudinal cohort study. Int J Env Res Pub He. (2022) 19(15):9198. 10.3390/ijerph19159198 PMC9367806 35954555

[24] Juhlin S Bergenheim A Gjertsson I Larsson A Mannerkorpi K. Physical activity with person-centred guidance supported by a digital platform for persons with chronic widespread pain: a randomized controlled trial. J Rehabil Med. (2021) 53(4):jrm00175. 10.2340/16501977-2796 33576434 PMC8814874

[25] Kim WD Shin D. Comparison of outcomes of physical therapy exercises combined with either a video-based smartphone application system or a written exercise program handout in 34 patients with non-specific neck pain. Med Sci Monit. (2024) 30:e945349. 10.12659/MSM.945349 39215449 PMC11373364

[26] Kloek CJJ Bossen D Spreeuwenberg PM Dekker J de Bakker DH Veenhof C. Effectiveness of a blended physical therapist intervention in people with hip osteoarthritis, knee osteoarthritis, or both: a cluster- randomized controlled trial. Phys Ther. (2018) 98(7):560–70. 10.1093/ptj/pzy045 29788253 PMC6016690

[27] Kloek CJJ van Tilburg ML Staal JB Veenhof C Bossen D. Development and proof of concept of a blended physiotherapeutic intervention for patients with non-specific low back pain. Physiotherapy. (2019) 105(4):483–91. 10.1016/j.physio.2018.12.006 31031023

[28] Krkoska P Vlazna D Sladeckova M Minarikova J Barusova T Batalik L. Adherence and effect of home-based rehabilitation with telemonitoring support in patients with chronic non-specific low back pain: a pilot study. Int J Environ Res Public Health. (2023) 20(2):1504. 10.3390/ijerph20021504 36674258 PMC9860722

[29] Lara-Palomo IC Antequera-Soler E Matarán-Peñarrocha GA Fernández-Sánchez M García-López H Castro-Sánchez AM. Comparison of the effectiveness of an e-health program versus a home rehabilitation program in patients with chronic low back pain: a double blind randomized controlled trial. Digit Health. (2022) 8(101690863):20552076221074482. 10.1177/20552076221074482 35111332 PMC8801654

[30] Li LC Sayre EC Xie H Clayton C Feehan LM. A community-based physical activity counselling program for people with knee osteoarthritis: feasibility and preliminary efficacy of the track-OA study. JMIR Mhealth Uhealth. (2017) 5(6):e86. 10.2196/mhealth.7863 28652228 PMC5504340

[31] Li LC Feehan LM Xie H Lu N Shaw C Gromala D. Efficacy of a physical activity counseling program with use of a wearable tracker in people with inflammatory arthritis: a randomized controlled trial. Arthritis Care Res (Hoboken). (2020) 72(12):1755–65. 10.1002/acr.24199 32248626

[32] Li LC Feehan LM Xie H Lu N Shaw CD Gromala D. Effects of a 12-week multifaceted wearable-based program for people with knee osteoarthritis: randomized controlled trial. JMIR Mhealth Uhealth. (2020) 8(7):e19116. 10.2196/19116 32618578 PMC7367519

[33] Lopéz-Marcos JJ Díaz-Arribas MJ Valera-Calero JA Navarro-Santana MJ Izquierdo-García J Ortiz-Gutiérrez RM. The added value of face-to-face supervision to a therapeutic exercise-based app in the management of patients with chronic low back pain: a randomized clinical trial. Sensors (Basel). (2024) 24(2):567. 10.3390/s24020567 38257659 PMC10819225

[34] Mbada CE Olaoye MI Dada OO Ayanniyi O Johnson OE Odole AC. Comparative efficacy of clinic-based and telerehabilitation application of mckenzie therapy in chronic low-back pain. Int J Telerehabil. (2019) 11(1):41–58. 10.5195/ijt.2019.6260 31341546 PMC6597146

[35] Nordin CA Michaelson P Gard G Eriksson MK. Effects of the web behavior change program for activity and multimodal pain rehabilitation: randomized controlled trial. J Med Internet Res. (2016) 18(10):e265. 10.2196/jmir.5634 27707686 PMC5071618

[36] Özden F Sari Z Karaman ÖN Aydoğmuş H. The effect of video exercise-based telerehabilitation on clinical outcomes, expectation, satisfaction, and motivation in patients with chronic low back pain. Ir J Med Sci. (2022) 191(3):1229–39. 10.1007/s11845-021-02727-8 34357527

[37] Özel M Kaya Ciddi P. The effectiveness of telerehabilitation-based structured exercise therapy for chronic nonspecific neck pain: a randomized controlled trial. J Telemed Telecare. (2024) 30(5):823–33. 10.1177/1357633X221095782 35570728

[38] Rafiq MT Abdul Hamid MS Hafiz E. The effect of rehabilitation protocol using mobile health in overweight and obese patients with knee osteoarthritis: a clinical trial. Adv Rheumatol (London, England). (2021) 61(1):63. 10.1186/s42358-021-00221-4 34689837

[39] Raiszadeh K Tapicer J Taitano L Wu J Shahidi B. In-Clinic versus web-based multidisciplinary exercise-based rehabilitation for treatment of low back pain: prospective clinical trial in an integrated practice unit model. J Med Internet Res. (2021) 23(3):e22548. 10.2196/22548 33734088 PMC8074858

[40] Ramalingam V Cheong SK Lee PF. Study of EEG alpha wave response on the effects of video-guided deep breathing on pain rehabilitation. Technol Health Care. (2023) 31(1):37–46. 10.3233/THC-213531 35723127

[41] Rodríguez Sánchez-Laulhé P Luque-Romero LG Barrero-García FJ Biscarri-Carbonero Á Blanquero J Suero-Pineda A. An exercise and educational and self-management program delivered with a smartphone app (CareHand) in adults with rheumatoid arthritis of the hands: randomized controlled trial. JMIR Mhealth Uhealth. (2022) 10(4):e35462. 10.2196/35462 35389367 PMC9030995

[42] Rodríguez Sánchez-Laulhé P Biscarri-Carbonero Á Suero-Pineda A Luque-Romero LG Barrero García FJ Blanquero J. The effects of a mobile app-delivered intervention in people with symptomatic hand osteoarthritis: a pragmatic randomized controlled trial. Eur J Phys Rehabil Med. (2023) 59(1):54–64. 10.23736/S1973-9087.22.07744-9 36633498 PMC10035439

[43] Sandal LF Bach K Øverås CK Svendsen MJ Dalager T Stejnicher Drongstrup Jensen J. Effectiveness of app-delivered, tailored self-management support for adults with lower back pain-related disability: a selfBACK randomized clinical trial. JAMA Intern Med. (2021) 181(10):1288–96. 10.1001/jamainternmed.2021.4097 34338710 PMC8329791

[44] Scheer J Costa F Molinos M Areias A Janela D Moulder RG. Racial and ethnic differences in outcomes of a 12-week digital rehabilitation program for musculoskeletal pain: prospective longitudinal cohort study. J Med Internet Res. (2022) 24(10):e41306. 10.2196/41306 36189963 PMC9664333

[45] Shi W Zhang Y Bian Y Chen L Yuan W Zhang H. The physical and psychological effects of telerehabilitation-based exercise for patients with nonspecific low back pain: prospective randomized controlled trial. JMIR Mhealth Uhealth. (2024) 12:e56580. 10.2196/56580 39240210 PMC11395168

[46] Toelle TR Utpadel-Fischler DA Haas K-K Priebe JA. App-based multidisciplinary back pain treatment versus combined physiotherapy plus online education: a randomized controlled trial. NPJ Digit Med. (2019) 2(101731738):34. 10.1038/s41746-019-0109-x 31304380 PMC6550294

[47] Uesugi Y Koyanagi J Takagi K Yamaguchi R Hayashi S Nishii T. Exercise therapy interventions in patients with hip osteoarthritis: comparison of the effects of DVD and website-based interventions. JMIR Rehabil Assist Technol. (2018) 20(5):e10. 10.2196/rehab.8251 PMC5962826 29735473

[48] Weber F Kloek C Stuhrmann S Blum Y Grüneberg C Veenhof C. Usability and preliminary effectiveness of an app-based physical activity and education program for people with hip or knee osteoarthritis—a pilot randomized controlled trial. Arthritis Res Ther. (2024) 26(1):83. 10.1186/s13075-024-03291-z 38600607 PMC11005282

[49] Weise H Zenner B Schmiedchen B Benning L Bulitta M Schmitz D. The Effect of an App-Based Home Exercise Program on Self-reported Pain Intensity in Unspecific and Degenerative Back Pain: Pragmatic Open-label Randomized Controlled Trial. (2022). 10.2196/41899 PMC9652727 36215327

[50] Yang J Wei Q Ge Y Meng L Zhao M. Smartphone-Based remote self-management of chronic low back pain: a preliminary study. J Healthc Eng. (2019) 2019(101528166):4632946. 10.1155/2019/4632946 30881606 PMC6381588

[51] Zheng F Zheng Y Liu S Yang J Xiao W Xiao W. The effect of M-health-based core stability exercise combined with self-compassion training for patients with nonspecific chronic low back pain: a randomized controlled pilot study. Pain Ther. (2022) 11(2):511–28. 10.1007/s40122-022-00358-0 35133634 PMC9098748

[52] Suzuki H Aono S Inoue S Imajo Y Nishida N Funaba M. Clinically significant changes in pain along the pain intensity numerical rating scale in patients with chronic low back pain. PLoS One. (2020) 15(3):e0229228. 10.1371/journal.pone.0229228 32126108 PMC7053735

[53] Stratford PW Riddle DL. A roland morris disability questionnaire target value to distinguish between functional and dysfunctional states in people with low back pain. Physiother Can. (2016) 68(1):29–35. 10.3138/ptc.2014-85 27504045 PMC4961316

[54] Power JD Perruccio AV Canizares M McIntosh G Abraham E Attabib N. Determining minimal clinically important difference estimates following surgery for degenerative conditions of the lumbar spine: analysis of the Canadian spine outcomes and research network (CSORN) registry. Spine J. (2023) 23(9):1323–33. 10.1016/j.spinee.2023.05.001 37160168

[55] Nishimoto J Kurahashi R Tamari K Tanaka R. Minimal clinically important difference in the hip disability and osteoarthritis outcome score (HOOS) for patients undergoing total hip arthroplasty: a multicenter prospective cohort study. J Orthop Surg (Hong Kong). (2026) 34(1):10225536261433423. 10.1177/10225536261433423 41925191

[56] Alsahli S Hor SY Lam M. Factors influencing the acceptance and adoption of Mobile health apps by physicians during the COVID-19 pandemic: systematic review. JMIR Mhealth Uhealth. (2023) 11:e50419. 10.2196/50419 37938873 PMC10666016

[57] Giebel GD Abels C Plescher F Speckemeier C Schrader NF Borchers K. Problems and barriers related to the use of mHealth apps from the perspective of patients: focus group and interview study. J Med Internet Res. (2024) 26:e49982. 10.2196/49982 38652508 PMC11077409

[58] Jacob C Sezgin E Sanchez-Vazquez A Ivory C. Sociotechnical factors affecting Patients’ adoption of Mobile health tools: systematic literature review and narrative synthesis. JMIR Mhealth Uhealth. (2022) 10(5):e36284. 10.2196/36284 35318189 PMC9121221

[59] Kong T Scott MM Li Y Wichelman C. Physician attitudes towards-and adoption of-mobile health. Digit Health. (2020) 6:2055207620907187. 10.1177/2055207620907187 32128235 PMC7036486

[60] Zhou T Salman D McGregor A. Mhealth apps for the self-management of low back pain: systematic search in app stores and content analysis. JMIR Mhealth Uhealth. (2024) 12:e53262. 10.2196/53262 38300700 PMC10870204

[61] George SZ Fritz JM Silfies SP Schneider MJ Beneciuk JM Lentz TA. Interventions for the management of acute and chronic low back pain: revision 2021. J Orthop Sports Phys Ther. (2021) 51(11):CPG1–CPG60. 10.2519/jospt.2021.0304 34719942 PMC10508241

[62] van Doormaal MCM Meerhoff GA Vliet Vlieland TPM Peter WF. A clinical practice guideline for physical therapy in patients with hip or knee osteoarthritis. Musculoskeletal Care. (2020) 18(4):575–95. 10.1002/msc.1492 32643252

[63] Pignato M Arbeeva L Schwartz TA Callahan LF Cooke J Golightly YM. Level of participation in physical therapy or an internet-based exercise training program: associations with outcomes for patients with knee osteoarthritis. BMC Musculoskelet Disord. (2018) 19(1):238. 10.1186/s12891-018-2139-y 30025540 PMC6053740

[64] Jensen MP Turner JA Romano JM Fisher LD. Comparative reliability and validity of chronic pain intensity measures. Pain. (1999) 83(2):157–62. 10.1016/S0304-3959(99)00101-3 10534586

[65] Fairbank JC Pynsent PB. The oswestry disability Index. Spine (Phila Pa 1976). (2000) 25(22):2940–52. discussion 52. 10.1097/00007632-200011150-00017 11074683

[66] Roland M Fairbank J. The roland-morris disability questionnaire and the oswestry disability questionnaire. Spine (Phila Pa 1976). (2000) 25(24):3115–24. 10.1097/00007632-200012150-00006 11124727

[67] McConnell S Kolopack P Davis AM. The western Ontario and McMaster universities osteoarthritis Index (WOMAC): a review of its utility and measurement properties. Arthritis Rheum. (2001) 45(5):453–61. 10.1002/1529-0131(200110)45:5<453::AID-ART365>3.0.CO;2-W 11642645

[68] Collins NJ Prinsen CA Christensen R Bartels EM Terwee CB Roos EM. Knee injury and osteoarthritis outcome score (KOOS): systematic review and meta-analysis of measurement properties. Osteoarthritis Cartilage. (2016) 24(8):1317–29. 10.1016/j.joca.2016.03.010 27012756

[69] Podsiadlo D Richardson S. The timed “up & go": a test of basic functional mobility for frail elderly persons. J Am Geriatr Soc. (1991) 39(2):142–8. 10.1111/j.1532-5415.1991.tb01616.x 1991946

[70] Herman T Giladi N Hausdorff JM. Properties of the ‘timed up and go’ test: more than meets the eye. Gerontology. (2011) 57(3):203–10. 10.1159/000314963 20484884 PMC3094679

[71] Guralnik JM Ferrucci L Pieper CF Leveille SG Markides KS Ostir GV. Lower extremity function and subsequent disability: consistency across studies, predictive models, and value of gait speed alone compared with the short physical performance battery. J Gerontol A Biol Sci Med Sci. (2000) 55(4):M221–31. 10.1093/gerona/55.4.M221 10811152 PMC12149745

[72] Guralnik JM Simonsick EM Ferrucci L Glynn RJ Berkman LF Blazer DG. A short physical performance battery assessing lower extremity function: association with self-reported disability and prediction of mortality and nursing home admission. J Gerontol. (1994) 49(2):M85–94. 10.1093/geronj/49.2.M85 8126356

[73] Rethorn ZD Pettitt CD. What is the effect of health coaching delivered by physical therapists? A systematic review of randomized controlled trials. Phys Ther. (2019) 99(10):1354–70. 10.1093/ptj/pzz098 31309976

[74] Prior JL Vesentini G Michell De Gregorio JA Ferreira PH Hunter DJ Ferreira ML. Health coaching for low back pain and hip and knee osteoarthritis: a systematic review with meta-analysis. Pain Med. (2023) 24(1):32–51. 10.1093/pm/pnac099 35775931 PMC9825146

[75] Morales ME Yong RJ. Racial and ethnic disparities in the treatment of chronic pain. Pain Med. (2021) 22(1):75–90. 10.1093/pm/pnaa427 33367911

[76] Zajacova A Lawrence EM. The relationship between education and health: reducing disparities through a contextual approach. Annu Rev Public Health. (2018) 39:273–89. 10.1146/annurev-publhealth-031816-044628 29328865 PMC5880718

[77] Dworkin RH Turk DC Wyrwich KW Beaton D Cleeland CS Farrar JT. Interpreting the clinical importance of treatment outcomes in chronic pain clinical trials: iMMPACT recommendations. J Pain. (2008) 9(2):105–21. 10.1016/j.jpain.2007.09.005 18055266

[78] Hopewell S Chan AW Collins GS Hrobjartsson A Moher D Schulz KF. CONSORT 2025 Statement: updated guideline for reporting randomized trials. JAMA. (2025) 333(22):1998–2005. 10.1001/jama.2025.4347 40228499

[79] Gewandter JS Eisenach JC Gross RA Jensen MP Keefe FJ Lee DA. Checklist for the preparation and review of pain clinical trial publications: a pain-specific supplement to CONSORT. Pain Rep. (2019) 4(3):e621. 10.1097/PR9.0000000000000621 28989992 PMC5625298

[80] Agarwal S LeFevre AE Lee J L'Engle K Mehl G Sinha C. Guidelines for reporting of health interventions using mobile phones: mobile health (mHealth) evidence reporting and assessment (mERA) checklist. Br Med J. (2016) 352:i1174. 10.1136/bmj.i1174 26988021

[81] Jakob R Harperink S Rudolf AM Fleisch E Haug S Mair JL. Factors influencing adherence to mHealth apps for prevention or management of noncommunicable diseases: systematic review. J Med Internet Res. (2022) 24(5):e35371. 10.2196/35371 35612886 PMC9178451

[82] Ratanawong JP Naslund JA Mikal JP Grande SW. Achieving the potential of mHealth in medicine requires challenging the ethos of care delivery. Prim Health Care Res Dev. (2022) 23:e18. 10.1017/S1463423622000068 35314016 PMC8991074

[83] Mescher T Hacker RL Martinez LA Morris CD Mishkind MC Garver-Apgar CE. Mobile health apps: guidance for evaluation and implementation by healthcare workers. J Technol Behav Sci. (2024) 10(2):224–35. 10.1007/s41347-024-00441-7

