# Supplementary material for: mHealth applications to enhance physical therapy outcomes among adults with chronic non-cancer pain: a scoping review
Source: Front Pain Res (Lausanne). 2026 Jun 11;7:1669079. doi: 10.3389/fpain.2026.1669079 (PMC13293871; doi:10.3389/fpain.2026.1669079)
Supplement: Supplementary file 1 [file Supplementaryfile1.docx]

**Appendix A. Database Search Strategies.**

All searches were originally run on February 11, 2022. An updated search was performed in Ovid MEDLINE on September 22, 2024 to find new publications.

1. Chronic Pain/

2. Pain management/

3. Arthralgia/ or Shoulder pain/

4. exp Back pain/

5. Headache/

6. Musculoskeletal Pain/ or Pelvic Girdle Pain/

7. Neck Pain/

8. Neuralgia/ or Causalgia/ or Piriformis Muscle Syndrome/ or Sciatica/

9. Pain, Postoperative/

10. Arthritis/ or Rotator Cuff Tear Arthropathy/

11. Osteoarthritis/

12. Arthritis, Rheumatoid/

13. Fibromyalgia/

14. Spinal stenosis/

15. Lupus Erythematosus, Systemic/

16. Migraine Disorders/

17. Myofascial Pain Syndromes/ or Temporomandibular Joint Dysfunction Syndrome/

18. Myalgia/

19. (arthralgia or arthros?s or arthrochondritis or arthrydonia or arthrosynovitis or articular pain* or aching joint* or arm pain* or extremity pain* or coxarthros?s or arthritides or arthritis or polyarthritides or polyarthritis or arthrosis deformans or rheumarthritis or rheumatism or fibrosit* or joint pain* or joint ache* or joint inflammation* or oligoarthritis* or polyarthralgia or back pain* or backpain* or backache* or back ache* or failed back surgery syndrome* or pelvic girdle pain* or causalgia or dorsalgia or chronic non?cancer pain* or cncp or myofascial pain* or extremity pain* or leg pain* or fibromyalgia* or fibrositi* or fibromyalgia-fibromyositis syndrome* or knee pain* or headache* or head ache* or cranial pain* or cephalgia* or cephalea* or cerebral pain* or cranialgia* or head pain* or musculoskeletal pain* or locomotor pain or muscular rheumatism or neckache* or neck ache* or neck pain* or neuralgia* or neuralgic pain* or neuralgy or nerve pain* or neurodynia* or neuropathy or neuropathic pain* or sciatica or cervical pain* or cervicalgi* or cervicodynia* or nociceptive pain* or somatic pain* or shoulder pain* or tissue pain* or whole body pain* or post-operative pain* or postoperative pain* or post operation pain* or post-surgical pain* or postsurgical pain* or referred pain* or osteoarthritis or osteoarthros?s or OA or osteoporos?s or pain management* or muscle pain* or myalgia or muscle tenderness or muscle tenderness or migraine* or hemicran* or lupus or libman-sacks disease or libman sacks disease or sacroiliitis or spin* stenosis or stenosis canalis or rotator cuff tear arthropathy or temporomandibular joint dysfunction syndrome).tw.

20. ((ach* or acute or chronic or dull or persistent or tingl* or split* or sharp or radiat* or crushing or suffering* or migratory or burn* or throb* or tender* or sore*) adj3 pain*).tw.

21. Physical Therapy Modalities/

22. Exercise Movement Techniques/

23. exp Exercise Therapy/

24. exp Musculoskeletal Manipulations/

25. (physical therap* or physical treatment* or neurophysiotherap* or physiotherap* or physio therap* or exercise therap* or exercise movement technique* or pilates based exercise* or pilates-based exercise* or pilates movement* or rehabilitation exercise* or remedial exercise* or manipulation therap* or musculoskeletal manipulation* or manipulative therap* or craniosacral massage* or reflexology or rolfing).tw.

26. Rehabilitation/

27. Activities of Daily Living/

28. Early Ambulation/

29. (rehabilitation or habilitation or activit* of daily living or early ambulation or readaption or readjustment or rehabilitative treatment* or revalidation).tw.

30. Exercise/

31. (exercise* or physical activit* or fitness training or fitness workout or physical conditioning or physical workout or physical work-out or physical conditioning).tw.

32. exp Cell phone/

33. (cell* phone* or car phone* or mobile phone* or cellular telephone* or mobile telephone* or iphone* or ipod* or android* or text messag* or texting* or smartphone* or smart phone* or short messag* service* or sms or multimedia messag* service* or mms).tw.

34. Mobile applications/ or Telemedicine/

35. (app or apps or mobile application* or mobile technolog* or electronic application*).tw.

36. (mobile health or telehealth or tele-health or ehealth or e-health or telemedicine or tele-medicine or mhealth or m-health or health coach* or virtual coach* or telemonitor*).tw.

37. Internet/

38. (internet or cyberspace or website* or world wide web or social media or social network*).tw.

39. Fitness tracker/

40. (fit bit* or fitbit* or smart watch* or smartwatch* or Apple watch* or fitness tracker* or ipad*).tw.

41. Computer-Assisted Instruction/

42. (elearn* or e-learn* or electronic learn* or electronic instruct* or electronic educat*).tw.

43. ((computer or online) adj2 (learn* or educat* or instruct*)).tw.

44. Electronic Mail/

45. (electronic mail or email* or e-mail* or electronic messag*).tw.

46. or/1-20

47. or/21-31

48. or/32-45

49. 46 and 47 and 48

Ovid Embase 1974 to 2022 February 10

1. Chronic pain/

2. Arthralgia/

3. Shoulder pain/

4. exp backache/

5. headache/

6. musculoskeletal pain/

7. pelvic girdle pain/

8. neck pain/

9. neuralgia/

10. complex regional pain syndrome type II/

11. piriformis syndrome/

12. sciatica/

13. postoperative pain/

14. arthritis/

15. rotator cuff tear arthropathy/

16. osteoarthritis/

17. rheumatoid arthritis/

18. fibromyalgia/

19. vertebral canal stenosis/

20. systemic lupus erythematosus/

21. migraine/

22. myofascial pain/

23. temporomandibular joint disorder/

24. myalgia/

25. (arthralgia or arthros?s or arthrochondritis or arthrydonia or arthrosynovitis or articular pain* or aching joint* or arm pain* or extremity pain* or coxarthros?s or arthritides or arthritis or polyarthritides or polyarthritis or arthrosis deformans or rheumarthritis or rheumatism or fibrosit* or joint pain* or joint ache* or joint inflammation* or oligoarthritis* or polyarthralgia or back pain* or backpain* or backache* or back ache* or failed back surgery syndrome* or pelvic girdle pain* or causalgia or dorsalgia or chronic non?cancer pain* or cncp or myofascial pain* or extremity pain* or leg pain* or fibromyalgia* or fibrositi* or fibromyalgia-fibromyositis syndrome* or knee pain* or headache* or head ache* or cranial pain* or cephalgia* or cephalea* or cerebral pain* or cranialgia* or head pain* or musculoskeletal pain* or locomotor pain or muscular rheumatism or neckache* or neck ache* or neck pain* or neuralgia* or neuralgic pain* or neuralgy or nerve pain* or neurodynia* or neuropathy or neuropathic pain* or sciatica or cervical pain* or cervicalgi* or cervicodynia* or nociceptive pain* or somatic pain* or shoulder pain* or tissue pain* or whole body pain* or post-operative pain* or postoperative pain* or post operation pain* or post-surgical pain* or postsurgical pain* or referred pain* or osteoarthritis or osteoarthros?s or OA or osteoporos?s or pain management* or muscle pain* or myalgia or muscle tenderness or muscle tenderness or migraine* or hemicran* or lupus or libman-sacks disease or libman sacks disease or sacroiliitis or spin* stenosis or stenosis canalis or rotator cuff tear arthropathy or temporomandibular joint dysfunction syndrome).tw.

26. ((ach* or acute or chronic or dull or persistent or tingl* or split* or sharp or radiat* or crushing or suffering* or migratory or burn* or throb* or tender* or sore*) adj3 pain*).tw.

27. or/1-26

28. physiotherapy/

29. exp kinesiotherapy/

30. exp musculoskeletal manipulation/

31. (physical therap* or physical treatment* or neurophysiotherap* or physiotherap* or physio therap* or exercise therap* or exercise movement technique* or pilates based exercise* or pilates-based exercise* or pilates movement* or rehabilitation exercise* or remedial exercise* or manipulation therap* or musculoskeletal manipulation* or manipulative therap* or craniosacral massage* or reflexology or rolfing).tw.

32. rehabilitation/

33. daily life activity/

34. mobilization/

35. (rehabilitation or habilitation or activit* of daily living or early ambulation or readaption or readjustment or rehabilitative treatment* or revalidation).tw.

36. exercise/

37. (exercise* or physical activit* or fitness training or fitness workout or physical conditioning or physical workout or physical work-out or physical conditioning).tw.

38. or/28-37

39. exp mobile phone/

40. (cell* phone* or car phone* or mobile phone* or cellular telephone* or mobile telephone* or iphone* or ipod* or android* or text messag* or texting* or smartphone* or smart phone* or short messag* service* or sms or multimedia messag* service* or mms).tw.

41. mobile application/

42. telemedicine/

43. (app or apps or mobile application* or mobile technolog* or electronic application*).tw.

44. (mobile health or telehealth or tele-health or ehealth or e-health or telemedicine or tele-medicine or mhealth or m-health or health coach* or virtual coach* or telemonitor*).tw.

45. Internet/

46. (internet or cyberspace or website* or world wide web or social media or social network*).tw.

47. activity tracker/

48. (fit bit* or fitbit* or smart watch* or smartwatch* or Apple watch* or fitness tracker* or iPad*).tw.

49. (computer assisted instruction or computer-assisted instruction or elearn* or e-learn* or electronic learn* or electronic instruct* or e-instruct* or electronic educat* or e-educat*).tw.

50. ((computer or online) adj2 (learn* or educat* or instruct*)).tw.

51. e-mail/

52. (electronic mail or email* or e-mail* or electronic messag*).tw.

53. or/39-52

54. 27 and 38 and 53

Cinahl (Ebsco)

S76 S73 AND S74 AND S75

S75 S58 OR S59 OR S60 OR S61 OR S62 OR S63 OR S64 OR S65 OR S66 OR S67 OR S68 OR S69 OR S70 OR S71 OR S72

S74 S49 OR S50 OR S51 OR S52 OR S53 OR S54 OR S55 OR S56 OR S57

S73 S1 OR S2 OR S3 OR S4 OR S6 OR S7 OR S8 OR S9 OR S10 OR S11 OR S12 OR S13 OR S14 OR S15 OR S16 OR S17 OR S18 OR S19 OR S20 OR S21 OR S22 OR S23 OR S28 OR S47 OR S48

S72 TI ( (electronic mail or email* or e-mail* or electronic messag*) ) OR AB ( (electronic mail or email* or e-mail* or electronic messag*) )

S71 (MH "Email")

S70 TI ( ((computer or online) N2 (learn* or educat* or instruct*)) ) OR AB ( ((computer or online) N2 (learn* or educat* or instruct*)) )

S69 TI ( (elearn* or e-learn* or electronic learn* or electronic instruct* or electronic educat*) ) OR AB ( (elearn* or e-learn* or electronic learn* or electronic instruct* or electronic educat*) )

S68 (MH "Computer Assisted Instruction")

S67 TI ( (fit bit* or fitbit* or smart watch* or smartwatch* or Apple watch* or fitness tracker* or ipad*) ) OR AB ( (fit bit* or fitbit* or smart watch* or smartwatch* or Apple watch* or fitness tracker* or ipad*) )

S66 (MH "Fitness Trackers")

S65 TI ( (internet or cyberspace or website* or world wide web or social media or social network*) ) OR AB ( (internet or cyberspace or website* or world wide web or social media or social network*) )

S64 (MH "Internet")

S63 TI ( (mobile health or telehealth or tele-health or ehealth or e-health or telemedicine or tele-medicine or mhealth or m-health or health coach* or virtual coach* or telemonitor*) ) OR AB ( (mobile health or telehealth or tele-health or ehealth or e-health or telemedicine or tele-medicine or mhealth or m-health or health coach* or virtual coach* or telemonitor*) )

S62 TI ( (app or apps or mobile application* or mobile technolog* or electronic application*) ) OR AB ( (app or apps or mobile application* or mobile technolog* or electronic application*) )

S61 (MH "Telemedicine")

S60 (MH "Mobile Applications")

S59 TI ( (cell* phone* or car phone* or mobile phone* or cellular telephone* or mobile telephone* or iphone* or ipod* or android* or text messag* or texting* or smartphone* or smart phone* or short messag* service* or sms or multimedia messag* service* or mms) ) OR AB ( (cell* phone* or car phone* or mobile phone* or cellular telephone* or mobile telephone* or iphone* or ipod* or android* or text messag* or texting* or smartphone* or smart phone* or short messag* service* or sms or multimedia messag* service* or mms) )

S58 (MH "Cellular Phone+")

S57 TI ( (exercise* or physical activit* or fitness training or fitness workout or physical conditioning or physical workout or physical work-out or physical conditioning) ) OR AB ( (exercise* or physical activit* or fitness training or fitness workout or physical conditioning or physical workout or physical work-out or physical conditioning) )

S56 (MH "Exercise")

S55 TI ( (rehabilitation or habilitation or activit* of daily living or early ambulation or readaption or readjustment or rehabilitative treatment* or revalidation) ) OR AB ( (rehabilitation or habilitation or activit* of daily living or early ambulation or readaption or readjustment or rehabilitative treatment* or revalidation) )

S54 (MH "Early Ambulation")

S53 (MH "Activities of Daily Living")

S52 (MH "Rehabilitation")

S51 TI ( (physical therap* or physical treatment* or neurophysiotherap* or physiotherap* or physio therap* or exercise therap* or exercise movement technique* or pilates based exercise* or pilates-based exercise* or pilates movement* or rehabilitation exercise* or remedial exercise* or manipulation therap* or musculoskeletal manipulation* or manipulative therap* or craniosacral massage* or reflexology or rolfing) ) OR AB ( (physical therap* or physical treatment* or neurophysiotherap* or physiotherap* or physio therap* or exercise therap* or exercise movement technique* or pilates based exercise* or pilates-based exercise* or pilates movement* or rehabilitation exercise* or remedial exercise* or manipulation therap* or musculoskeletal manipulation* or manipulative therap* or craniosacral massage* or reflexology or rolfing) )

S50 (MH "Therapeutic Exercise+")

S49 (MH "Physical Therapy")

S48 TI ( ((ach* or acute or chronic or dull or persistent or tingl* or split* or sharp or radiat* or crushing or suffering* or migratory or burn* or throb* or tender* or sore*) N3 (pain*) ) OR AB ( ((ach* or acute or chronic or dull or persistent or tingl* or split* or sharp or radiat* or crushing or suffering* or migratory or burn* or throb* or tender* or sore*) N3 (pain*) )

S47 TI ( (arthralgia or arthros?s or arthrochondritis or arthrydonia or arthrosynovitis or articular pain* or aching joint* or arm pain* or extremity pain* or coxarthros?s or arthritides or arthritis or polyarthritides or polyarthritis or arthrosis deformans or rheumarthritis or rheumatism or fibrosit* or joint pain* or joint ache* or joint inflammation* or oligoarthritis* or polyarthralgia or back pain* or backpain* or backache* or back ache* or failed back surgery syndrome* or pelvic girdle pain* or causalgia or dorsalgia or chronic non?cancer pain* or cncp or myofascial pain* or extremity pain* or leg pain* or fibromyalgia* or fibrositi* or fibromyalgia-fibromyositis syndrome* or knee pain* or headache* or head ache* or cranial pain* or cephalgia* or cephalea* or cerebral pain* or cranialgia* or head pain* or musculoskeletal pain* or locomotor pain or muscular rheumatism or neckache* or neck ache* or neck pain* or neuralgia* or neuralgic pain* or neuralgy or nerve pain* or neurodynia* or neuropathy or neuropathic pain* or sciatica or cervical pain* or cervicalgi* or cervicodynia* or nociceptive pain* or somatic pain* or shoulder pain* or tissue pain* or whole body pain* or post-operative pain* or postoperative pain* or post operation pain* or post-surgical pain* or postsurgical pain* or referred pain* or osteoarthritis or osteoarthros?s or OA or osteoporos?s or pain management* or muscle pain* or myalgia or muscle tenderness or muscle tenderness or migraine* or hemicran* or lupus or libman-sacks disease or libman sacks disease or sacroiliitis or spin* stenosis or stenosis canalis or rotator cuff tear arthropathy or temporomandibular joint dysfunction syndrome) ) OR AB ( (arthralgia or arthros?s or arthrochondritis or arthrydonia or arthrosynovitis or articular pain* or aching joint* or arm pain* or extremity pain* or coxarthros?s or arthritides or arthritis or polyarthritides or polyarthritis or arthrosis deformans or rheumarthritis or rheumatism or fibrosit* or joint pain* or joint ache* or joint inflammation* or oligoarthritis* or polyarthralgia or back pain* or backpain* or backache* or back ache* or failed back surgery syndrome* or pelvic girdle pain* or causalgia or dorsalgia or chronic non?cancer pain* or cncp or myofascial pain* or extremity pain* or leg pain* or fibromyalgia* or fibrositi* or fibromyalgia-fibromyositis syndrome* or knee pain* or headache* or head ache* or cranial pain* or cephalgia* or cephalea* or cerebral pain* or cranialgia* or head pain* or musculoskeletal pain* or locomotor pain or muscular rheumatism or neckache* or neck ache* or neck pain* or neuralgia* or neuralgic pain* or neuralgy or nerve pain* or neurodynia* or neuropathy or neuropathic pain* or sciatica or cervical pain* or cervicalgi* or cervicodynia* or nociceptive pain* or somatic pain* or shoulder pain* or tissue pain* or whole body pain* or post-operative pain* or postoperative pain* or post operation pain* or post-surgical pain* or postsurgical pain* or referred pain* or osteoarthritis or osteoarthros?s or OA or osteoporos?s or pain management* or muscle pain* or myalgia or muscle tenderness or muscle tenderness or migraine* or hemicran* or lupus or libman-sacks disease or libman sacks disease or sacroiliitis or spin* stenosis or stenosis canalis or rotator cuff tear arthropathy or temporomandibular joint dysfunction syndrome) )

S46 (MH "Muscle Pain")

S45 (MH "Myofascial Pain Syndromes") OR (MH "Temporomandibular Joint Syndrome")

S44 (MH "Migraine")

S43 (MH "Lupus Erythematosus, Systemic")

S42 (MH "Spinal Stenosis")

S41 (MH "Fibromyalgia")

S40 (MH "Arthritis, Rheumatoid")

S39 (MH "Osteoarthritis")

S38 (MH "Rotator Cuff Injuries")

S37 (MH "Arthritis")

S36 (MH "Postoperative Pain")

S35 (MH "Sciatica")

S34 (MH "Piriformis Syndrome")

S33 (MH "Causalgia")

S32 (MH "Neuralgia")

S31 (MH "Neck Pain")

S30 (MH "Pelvic Pain")

S29 (MH "Headache") -

S28 (MH "Back Pain+") OR (MH "Low Back Pain")

S27 (MH "Shoulder Pain")

S26 (MH "Arthralgia")

S25 (MH "Pain Management")

S24 (MH "Chronic Pain")

S23 (MH "Muscle Pain")

S22 (MH "Myofascial Pain Syndromes") OR (MH "Temporomandibular Joint Syndrome")

S21 (MH "Migraine")

S20 (MH "Lupus Erythematosus, Systemic")

S19 (MH "Spinal Stenosis")

S18 (MH "Fibromyalgia")

S17 (MH "Arthritis, Rheumatoid")

S16 (MH "Osteoarthritis")

S15 (MH "Rotator Cuff Injuries")

S14 (MH "Arthritis")

S13 (MH "Postoperative Pain")

S12 (MH "Sciatica")

S11 (MH "Piriformis Syndrome")

S10 (MH "Causalgia")

S9 (MH "Neuralgia")

S8 (MH "Neck Pain")

S7 (MH "Pelvic Pain")

S6 (MH "Headache")

S5 (MH "Back Pain") OR (MH "Low Back Pain")

S4 (MH "Shoulder Pain")

S3 (MH "Arthralgia")

S2 (MH "Pain Management")

S1 (MH "Chronic Pain")

Cochrane Library (Wiley)

#1 MeSH descriptor: [Chronic Pain] this term only

#2 MeSH descriptor: [Pain Management] this term only

#3 MeSH descriptor: [Arthralgia] this term only

#4 MeSH descriptor: [Shoulder Pain] this term only

#5 MeSH descriptor: [Back Pain] explode all trees

#6 MeSH descriptor: [Headache] this term only

#7 MeSH descriptor: [Musculoskeletal Pain] this term only

#8 MeSH descriptor: [Pelvic Girdle Pain] this term only

#9 MeSH descriptor: [Neck Pain] this term only

#10 MeSH descriptor: [Neuralgia] this term only

#11 MeSH descriptor: [Causalgia] this term only

#12 MeSH descriptor: [Piriformis Muscle Syndrome] this term only

#13 MeSH descriptor: [Sciatica] this term only

#14 MeSH descriptor: [Pain, Postoperative] this term only

#15 MeSH descriptor: [Arthritis] this term only

#16 MeSH descriptor: [Rotator Cuff Tear Arthropathy] this term only

#17 MeSH descriptor: [Osteoarthritis] this term only

#18 MeSH descriptor: [Arthritis, Rheumatoid] this term only

#19 MeSH descriptor: [Fibromyalgia] this term only

#20 MeSH descriptor: [Spinal Stenosis] this term only

#21 MeSH descriptor: [Lupus Erythematosus, Systemic] this term only

#22 MeSH descriptor: [Migraine Disorders] this term only

#23 MeSH descriptor: [Myofascial Pain Syndromes] this term only

#24 MeSH descriptor: [Temporomandibular Joint Dysfunction Syndrome] this term only

#25 MeSH descriptor: [Myalgia] this term only

#26 ((arthralgia or arthros?s or arthrochondritis or arthrydonia or arthrosynovitis or articular pain* or aching joint* or arm pain* or extremity pain* or coxarthros?s or arthritides or arthritis or polyarthritides or polyarthritis or arthrosis deformans or rheumarthritis or rheumatism or fibrosit* or joint pain* or joint ache* or joint inflammation* or oligoarthritis* or polyarthralgia or back pain* or backpain* or backache* or back ache* or failed back surgery syndrome* or pelvic girdle pain* or causalgia or dorsalgia or chronic non?cancer pain* or cncp or myofascial pain* or extremity pain* or leg pain* or fibromyalgia* or fibrositi* or fibromyalgia-fibromyositis syndrome* or knee pain* or headache* or head ache* or cranial pain* or cephalgia* or cephalea* or cerebral pain* or cranialgia* or head pain* or musculoskeletal pain* or locomotor pain or muscular rheumatism or neckache* or neck ache* or neck pain* or neuralgia* or neuralgic pain* or neuralgy or nerve pain* or neurodynia* or neuropathy or neuropathic pain* or sciatica or cervical pain* or cervicalgi* or cervicodynia* or nociceptive pain* or somatic pain* or shoulder pain* or tissue pain* or whole body pain* or post-operative pain* or postoperative pain* or post operation pain* or post-surgical pain* or postsurgical pain* or referred pain* or osteoarthritis or osteoarthros?s or OA or osteoporos?s or pain management* or muscle pain* or myalgia or muscle tenderness or muscle tenderness or migraine* or hemicran* or lupus or libman-sacks disease or libman sacks disease or sacroiliitis or spin* stenosis or stenosis canalis or rotator cuff tear arthropathy or temporomandibular joint dysfunction syndrome)):ti,ab 199877

#27 ((ach* or acute or chronic or dull or persistent or tingl* or split* or sharp or radiat* or crushing or suffering* or migratory or burn* or throb* or tender* or sore*) NEAR/3 (pain*)):ti,ab

#28 #1 OR #2 or #3 OR #4 OR #5 OR #6 OR #7 OR #8 OR #9 OR #10 OR #11 OR #12 OR #13 OR #14 OR #15 OR #16 OR #17 OR #18 OR #19 OR #20 OR #21 OR #22 OR #23 OR #24 OR #25 OR #26 OR #27

#29 MeSH descriptor: [Physical Therapy Modalities] this term only

#30 MeSH descriptor: [Exercise Movement Techniques] this term only

#31 MeSH descriptor: [Exercise Therapy] explode all trees

#32 MeSH descriptor: [Musculoskeletal Manipulations] explode all trees

#33 (physical therap* or physical treatment* or neurophysiotherap* or physiotherap* or physio therap* or exercise therap* or exercise movement technique* or pilates based exercise* or pilates-based exercise* or pilates movement* or rehabilitation exercise* or remedial exercise* or manipulation therap* or musculoskeletal manipulation* or manipulative therap* or craniosacral massage* or reflexology or rolfing):ti,ab

#34 MeSH descriptor: [Rehabilitation] this term only

#35 MeSH descriptor: [Activities of Daily Living] this term only

#36 MeSH descriptor: [Early Ambulation] this term only

#37 (rehabilitation or habilitation or activit* of daily living or early ambulation or readaption or readjustment or rehabilitative treatment* or revalidation):ti,ab

#38 MeSH descriptor: [Exercise] this term only

#39 (exercise* or physical activit* or fitness training or fitness workout or physical conditioning or physical workout or physical work-out or physical conditioning):ti,ab

#40 #29 OR #30 OR #31 OR #32 OR #33 OR #34 OR #35 OR #36 OR #37 OR #38 OR #39

#41 MeSH descriptor: [Cell Phone] explode all trees

#42 (cell* phone* or car phone* or mobile phone* or cellular telephone* or mobile telephone* or iphone* or ipod* or android* or text messag* or texting* or smartphone* or smart phone* or short messag* service* or sms or multimedia messag* service* or mms):ti,ab

#43 MeSH descriptor: [Mobile Applications] this term only

#44 MeSH descriptor: [Telemedicine] this term only

#45 (app or apps or mobile application* or mobile technolog* or electronic application*):ti,ab

#46 (mobile health or telehealth or tele-health or ehealth or e-health or telemedicine or tele-medicine or mhealth or m-health or health coach* or virtual coach* or telemonitor*):ti,ab

#47 MeSH descriptor: [Internet] this term only

#48 (internet or cyberspace or website* or world wide web or social media or social network*):ti,ab

#49 MeSH descriptor: [Fitness Trackers] this term only

#50 (fit bit* or fitbit* or smart watch* or smartwatch* or Apple watch* or fitness tracker* or ipad* ):ti,ab

#51 MeSH descriptor: [Computer-Assisted Instruction] this term only

#52 (elearn* or e-learn* or electronic learn* or electronic instruct* or e-instruct* or electronic educat* or e-educat*):ti,ab

#53 ((computer or online) NEAR/2 (learn* or educat* or instruct*)):ti,ab

#54 MeSH descriptor: [Electronic Mail] this term only

#55 (electronic mail or email* or e-mail* or electronic messag*):ti,ab

#56 #41 OR #42 OR #43 OR #44 OR #45 OR #46 OR #47 OR #48 OR #49 OR #50 OR #51 OR #52 OR #53 OR #54 OR #55 53548

#57 #28 AND #40 AND #56
