# Supplementary material for: mHealth applications to enhance physical therapy outcomes among adults with chronic non-cancer pain: a scoping review
Source: Front Pain Res (Lausanne). 2026 Jun 11;7:1669079. doi: 10.3389/fpain.2026.1669079 (PMC13293871; doi:10.3389/fpain.2026.1669079)
Supplement: Supplementary file 2 [file Supplementaryfile2.docx]

**Appendix B. Scoping variables**

| **Covidence #** |
| --- |
| **Study ID** |
| **Title** |
| **Reviewer Name** |
| **Manuscript title** |
| **Article number** |
| **First author** |
| **Country/region in which study conducted** |
| **Country/region code** |
| **Funding Source** |
| **Start Date** |
| **End Date** |
| **Type of study** |
| **Primary study purpose** |
| **Secondary purpose** |
| **Study Design** |
| **Type of mHealth Intervention** |
| **Is there evidence that training was provided to participants?** |
| **Was ongoing support provided** |
|  |
| **Content of mHealth Intervention** |
| **Additional interventions for mHealth group** |
| **Control Group Intervention** |
|  |
| **Frequency of mHealth interventions** |
| **Recruitment setting** |
|  |
| **Number of subjects per group** |
| **Total N** |
| **Participant diagnosis** |
| **Pain location (if inclusion criterion)** |
| **Other inclusion criteria** |
| **Exclusion criteria** |
| **Inclusion/Exclusion criteria additional comments** |
| **Intervention length** |
|  |
| **Timepoints for outcomes** |
| **Adherence measured (Y/N)** |
| **Primary Outcome specified?** |
|  |
| **Primary outcome** |
|  |
| **Pain outcome measured** |
| **Standardized Pain Instrument; if used** |
| **Functional outcomes (list)** |
| **Psychological outcomes (list)** |
|  |
| **Demographics** |
| **Age mHealth** |
| **Age Control** |
| **Age Total** |
| **Gender/sex mHealth** |
| **Gender/sex Control** |
| **Gender/sex Total** |
| **Race/Ethnicity mHealth** |
| **Race/Ethnicity Control** |
| **Race/Ethnicity Total** |
| **Was educational level of participants reported?** |
| **Results at end of intervention period:** |
| **Pain mHealth** |
| **Pain Control** |
| **Function mHealth** |
| **Function Control** |
| **Psychological state mHealth** |
| **Psychological state Control** |
|  |
| **Intervention adherence mHealth** |
| **Intervention adherence Control** |
| **Study retention (%) mHealth** |
|  |
| **Study retention (%) Control** |
| **Other standardized assessment mHealth** |
| **Other standardized assessment Control** |
| **mHealth** |
| **Control** |
| **Indicate general outcomes at additional timepoints** |
|  |
| **Was the incidence (or lack) of adverse events reported?** |
| **Was the number of subjects lost to follow-up reported?** |
| **Additional Comments** |
|  |
|  |
|  |
